# An Integrated Low-Cost Underwater Navigation Solution for Divers Employing an INS Composed of Low-Cost Sensors Using the Robust Kalman Filter and Sensor Fusion

**DOI:** 10.3390/s25185750

**Published:** 2025-09-15

**Authors:** Taisei Hayashi, Daisuke Terada

**Affiliations:** Department of Mechanical Systems Engineering, National Defense Academy of Japan, Hashirimizu 1-10-20, Yokosuka 239-8686, Kanagawa, Japan

**Keywords:** nautical instruments, underwater navigation, low cost IMU, quaternion, Robust Kalman Filter

## Abstract

Divers’ navigation heavily depends on their experience and physical condition, and accidents caused by failure to return occur every year. To address this issue, we developed a navigation system for divers. This navigation system leverages Raspberry Pi and low-cost sensors, including an accelerometer, gyro sensor, geomagnetic sensor, and pressure gauge, to guide divers along predefined routes back to their starting point. The system employs a 20 Hz sampling frequency and applies high-pass filtering (HPF) to acceleration signals to eliminate gravitational interference. Velocity integration errors are corrected using the rate of pressure change, while impulse noise in accelerometer and geomagnetic sensors is removed via the Robust Kalman Filter (RKF). A time-varying system noise covariance matrix enhances accuracy during rotational states. Quaternion-based attitude avoids gimbal lock, with the Kalman Filter (KF) fusion of accelerometer/geomagnetic data mitigating gyro sensor drift. Forced oscillator trials achieved pitch/roll RMS errors of ±1.23° and ±0.26°. In Kanagawa, Japan, divers successfully navigated 44 waypoints (<5 m spacing) along a route with obstacles (30 m rope, Authors, reefs), with a start/end GNSS positioning error of 6.67 m.

## 1. Introduction

Divers commonly estimate their position by measuring their travel direction and elapsed time. The reliability of this method depends on the diver’s experience and physical condition, and it requires advanced skills and knowledge. In 2023, 48 scuba diving incidents were reported in Japan, with “failure to return” being the third most common cause. When categorized by diving experience, the highest number of incidents occurred among divers with 100–500 dives [1]. These findings suggest that providing divers with a device capable of accurately estimating their position could reduce disorientation, particularly for semi-professional divers with 100–500 dives. In terrestrial environments, low-cost inertial navigation systems (INSs) in conjunction with Global Navigation Satellite Systems (GNSSs), analogous to those utilized in smartphones, are frequently employed. However, underwater, GNSS signals attenuate rapidly with depth and are often unavailable. On the other hand, underwater, GNSS signals attenuate with depth, and pressure changes with depth are greater than on land. Humans must wear extensive equipment to operate underwater, which often limits hand movement and visibility. For GNSS-denied platforms, highly reliable sensors and external information—such as data from a support vessel—are essential. As external sources, sonar is commonly utilized to detect, identify, and measuring distances to underwater objects [2] or information from a support vessel, and data obtained upon surfacing can be utilized [3]. In the field of underwater navigation, much research is being conducted on unmanned underwater vehicles (UUVs). UUV localization methods can be broadly classified into three categories: signal-based navigation, dead reckoning, and map-matching navigation [4]. Signal-based navigation relies on GNSS or sonar and provides high accuracy without cumulative error. Acoustic signal-based navigation can be divided into Super Short Baseline (SSBL), Short Baseline (SBL), and Long Baseline (LBL) [5]. SSBL and SBL use a single transponder with three or more receivers [5]. Achieving higher accuracy requires wider receiver spacing, which makes these methods suitable for large vessels. In contrast, LBL uses a single receiver with three or more transponders [5] and is not limited by receiver spacing. Dead reckoning utilizes IMUs and velocity sensors. Although it produces stable outputs, cumulative errors increase over time [6]. Ishibashi et al. [7] proposed mitigating these errors by rotating the INS. Map-matching navigation uses seafloor maps or onboard cameras. It is self-contained and free from cumulative error, but its accuracy depends heavily on environmental conditions [4]. Camera-based methods include Visual Odometry (VO) and Visual Simultaneous Localization and Mapping (V-SLAM) [8]. VO, sometimes regarded as a subset of SLAM, sequentially estimates the camera’s pose and the surrounding environment using only image data. A major limitation is that cameras without depth estimation cannot accurately measure displacement [9]. Forster et al. [10] addressed this by increasing frame rates and detecting outliers. Taguchi et al. [9] applied transfer learning for depth estimation. Liu et al. [11] employed multi-camera systems. V-SLAM estimates the platform’s position while simultaneously building a map [8]. LiDAR-SLAM, which combines LiDAR-based map-matching with dead reckoning, is becoming increasingly common in UUVs [8]. Map-matching is especially effective in shallow waters with known terrain, but its performance depends on light penetration. Somajyoti et al. [12] proposed integrating signal-based and map-matching navigation. However, the equipment required for these methods is generally expensive and impractical for individual divers. Divers also carry multiple devices and have limited mobility underwater, and cables or bulky equipment can compromise safety. Therefore, compact and low-cost navigation devices are needed to ensure underwater safety. Currently, no such system exists for divers. A device enabling navigation in unfamiliar dive sites could reduce mental stress and lower the risk of accidents. If such a system were inexpensive, it could achieve widespread adoption. One approach is signal-based navigation, a diver’s navigation system. It estimates position using a reference signal attached to the vessel and the diver’s equipment. However, these are expensive and cannot be used in places that vessels cannot access, such as beaches. Another approach is to build an INS using low-cost MEMS sensors. However, these sensors have a low signal-to-noise ratio (SNR), requiring appropriate signal processing. To address this, Hirose et al. [13] proposed a method that utilizes accelerometers, gyro sensors, and geomagnetic sensors with the Kalman Filter (KF) for correction. This method, however, relies heavily on accelerometer and geomagnetic sensor accuracy and does not address magnetic disturbances, such as those caused by nearby ferromagnetic materials. To correct magnetic disturbances, Kaiqiang et al. [14] utilized accelerometer data, but low-cost accelerometers are prone to outliers, limiting the practicality of this approach. Furthermore, since the values of geomagnetic sensors vary greatly depending on the direction they are facing, if they change direction frequently, they must be treated as a time-varying system. For outlier suppression, such as spike noise, the Robust Kalman Filter (RKF) is more effective. For attitude estimation, Euler angles or quaternions are commonly utilized. Euler angles are prone to gimbal lock, whereas quaternions avoid this problem but are less intuitive for human interpretation. For instance, Joan [15] employed both representations simultaneously, but the benefits were not fully realized because the computations ultimately relied on the Euler angle rotation matrix. These issues can be summarized as follows:

1. Navigation on land relies heavily on GNSS, which is not utilized underwater due to the attenuation of GNSS signals with depth.
2. It is challenging for human divers to navigate while tethered by cables or carrying substantial equipment, as this compromises safety.
3. Existing acoustic-based navigation systems for divers are prohibitively expensive and cannot be utilized on beaches where ships cannot navigate.
4. Sensor fusion for attitude estimation generally involves removing gyro sensor drift errors in the C-frame. However, when using low-SNR sensors, such as low-cost accelerometers and geomagnetic sensors, it is difficult to distinguish between noise and integration drift at the C-frame stage. Therefore, it is necessary to preprocess accelerometer and geomagnetic sensor signals in the S-frame and perform drift correction there, allowing noise and drift errors to be addressed separately.
5. The approach of using an accelerometer to correct magnetic disturbances from a geomagnetic sensor is difficult with low-SNR accelerometers.
6. When the attitude changes frequently, the geomagnetic sensor’s values fluctuate significantly depending on the direction, so it should be modeled as a time-varying system with an aspect that has not yet been addressed.
7. Quaternion-based attitude estimation cannot fully utilize its advantages when relying on Euler angles for continuous attitude changes, and low-SNR sensors cannot accurately estimate attitude when corrections are applied in a translational coordinate system.

This study aims to develop an underwater navigation system that utilizes low-cost sensors to enable divers to return to their departure point via a predetermined route, thereby addressing these issues. As to the attitude estimation, we extend our previous study [16] and replaced the analog attitude estimator with an acceleration-based attitude estimator. The characteristics of this study are as follows:

1. To address Problem 1, we utilize depth changes measured by a water pressure gauge. Because water pressure varies significantly with depth underwater compared to on land, even a low-cost pressure gauge can accurately estimate depth to within a few centimeters. We utilize this feature to correct the integration error from acceleration to velocity.
2. To address Problems 2 and 3, we propose a navigation system designed to be held in one hand.
3. To address Problem 4, we first preprocess the accelerometer and geomagnetic sensor signals using the RKF. Subsequently, sensor fusion is performed in the S-frame using a KF. Finally, the estimated attitude is transformed into the C-frame using quaternions.
4. To address Problems 5 and 6, we apply the RKF that models the geomagnetic sensor as a time-varying system and adapts system noise according to changes in the gyro sensor’s *z*-axis. This approach allows accurate azimuth estimation in magnetically disturbed environments and outperforms the conventional KF under transient conditions.
5. To address Problem 7, signals are processed in the S-frame and converted to quaternions. The quaternion is then transformed into Euler angles only for user display of the azimuth angle, thereby helping to avoid gimbal lock.

In Section 1, the background and objectives of this paper are discussed. In Section 2, the coordinate system used in this paper is presented. In Section 3, an overview of the proposed system is provided. In Section 4, the signal processing methods are explained in detail. In Section 5, the experimental results and discussions are presented. Finally, in Section 6, the obtained findings are summarized.

## 2. Coordinate System

**Figure 1:**
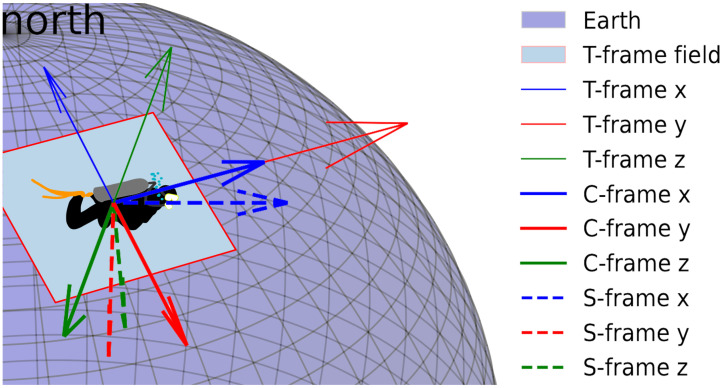
Coordinate systems.

In this study, three types of coordinate systems (shown in Figure 1) are used. The T-frame is in the East–North–Up (ENU) coordinate system, the C-frame has a rotation-relative coordinate system, and the S-frame is a right-hand side system with the *z* axis pointing downward.

## 3. Proposed System

The proposed system consists of Raspberry Pi manufactured by Raspberry Pi Ltd. (England, United Kingdom) and low-cost sensors, which are accelerometer and gyro sensor (MUP9250) manufactured by TDK Co., Ltd. (Tokyo, Japan), geomagnetic sensor (QMC5883L) manufactured by QST Co., Ltd. (Shanghai, China), and pressure gauge (MS5837-30BA) manufactured by TE Connectivity (Berwyn, Pennsylvania, United States) An accelerometer set scale ±2 g with SSF 16384 LSB/g [17]. A gyro sensor set scale ±250 deg/s with SSF 131 LSB/(deg/s) [17]. A geomagnetic sensor set scale ±8 G with SSF 3000 LSB/G [18]. The pressure gauge accuracy is ±1.5 mbr [19], which translates to ±150 pa.

First, to understand the diving motion frequency, it was recorded at a sampling frequency of 100 Hz. Since the dominant frequency of the motion was below 10 Hz as a result of the spectral analysis for the acceleration, the sampling frequency was decided to be 20 Hz. Next, characteristics of sensors in the steady state while the diver was at rest were investigated for calibration procedures and signal processing. For the accelerometer, the frequency band below 1 Hz dominated with spectral analysis. To address the influence of gravity and these low-frequency components, the HPF was applied to the signal processing of the accelerometer. HPF cut-off frequency was set to 0.8 Hz. As mentioned previously, the sensor results have frequent spike noise; thus, RKF was applied to remove it. For the gyro sensor, a dominant frequency did not appear.

**Figure 2:**
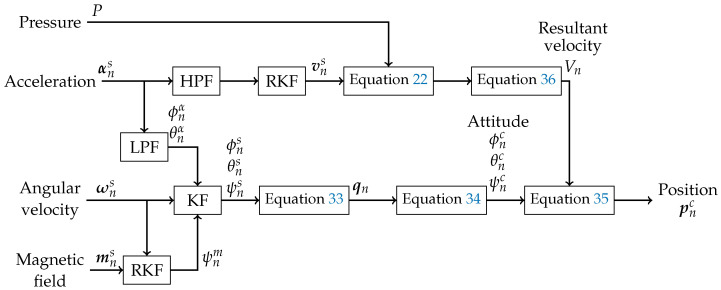
Process flow of proposed system.

Figure 2 shows the process flow of the proposed system. The process flow can be broadly divided into two steps. The first is estimating the resultant velocity, and the other is estimating the attitude. To estimate the resultant velocity, the aforementioned investigation function processes signals using the accelerometer and the RKF to determine the speed. The effect of the integral error included in the velocity derived by the RKF was removed by referring to the value of the pressure gauge.

In attitude estimation, gyro sensor outputs have drift and integration errors. While the geomagnetic sensor readings exhibit robustness, they necessitate consideration of rotational attitude when the device is not horizontal. Additionally, magnetic disturbances must be considered. To address them, the gyro sensor values integrated in the S-frame are corrected by the KF using the acceleration processed by the HPF and the heading angle of the geomagnetic sensor processed by the RKF. The rectified signal is converted to a quaternion-based attitude. The position is converted to the T-frame and is expressed with the latitude and longitude. The velocity components in the C-frame *x* and *y* axes are combined, and a coordinate transformation is performed using the ψ angle of the quaternion attitude corrected by the geomagnetic sensor.

**Figure 3:**
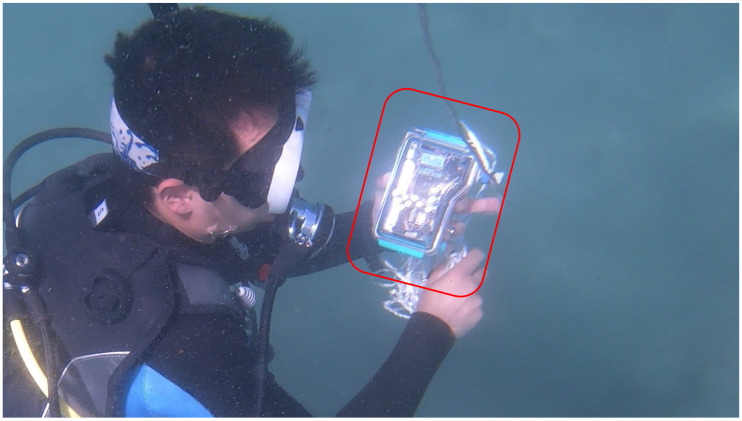
External appearance of the equipment incorporating the proposed system, as indicated by the red circle in the figure.

Finally, waypoints are set at intervals within 5 m to eliminate integration errors from velocity to position. Based on this, guidance is displayed to the user to show the direction and distance to advance. Figure 3 shows the external appearance of the equipment incorporating the proposed system.

## 4. Signal Processing

### 4.1. Drift Correction Using the Kalman Filter for Gyro Sensor [13]

Let the state x = ${\left[\phi_{n}^{s},\theta_{n}^{s},\psi_{n}^{s}\right]}^{T}$, the observation $y={\left[\phi_{n}^{\alpha },\theta_{n}^{\alpha },\psi_{n}^{m}\right]}^{T}$, and the input $u={\left[\omega_{x},\omega_{y},\omega_{z}\right]}^{T}$. Its state–space model is represented by the following Equation (1). Here, the range of $\phi_{n}^{\alpha }$ and $\theta_{n}^{\alpha }$ is from −π/2 to π/2.

(1) $$\left\{\begin{array}{c} x_{n}=x_{n-1}+\Delta tu_{n}+v_{n}\\ y_{n}=x_{n}+w_{n} \end{array}\right.$$

where $v_{n}$ is the system noise vector and is the three-dimensional Gaussian white noise sequence N(0,Q). Q is the variance–covariance matrix of the observation noise expressed as follows:

(2) $$\begin{array}{c} Q=\frac{\Delta t}{2}\mathrm{diag}\left(\sigma_{x}^{2},\sigma_{y}^{2},\sigma_{z}^{2}\right) \end{array}$$

$w_{n}$ is the observation noise vector and is the three-dimensional Gaussian white noise sequence N(0,R). R is a variance–covariance matrix of observation noise, and is expressed as follows:

(3) $$R=\left[\begin{array}{ccc} \sigma_{x}^{2} & \sigma_{xy} & \sigma_{xz}\\ \sigma_{xy} & \sigma_{y}^{2} & \sigma_{yz}\\ \sigma_{xz} & \sigma_{yz} & \sigma_{z}^{2} \end{array}\right]$$

Here, each element of this matrix is calculated from sensor data while the sensor is in a stationary state. The state estimation of Equation (1) can be completed by the algorithm of the KF shown in Equations (4)–(8).

[One-step-ahead prediction]

(4) $$\begin{array}{cc} \; & {\hat{x}}_{n|n-1}={\hat{x}}_{n-1|n-1}+\Delta tu_{n} \end{array}$$

(5) $$\begin{array}{cc} \; & P_{n|n-1}=P_{n-1|n-1}+Q \end{array}$$

[Filtering]

(6) $$\begin{array}{cc} \; & {\hat{x}}_{n|n}={\hat{x}}_{n|n-1}+K_{n}\left(y_{n}-{\hat{x}}_{n|n-1}\right) \end{array}$$

(7) $$\begin{array}{cc} \; & P_{n|n}=\left(I-K_{n}\right)P_{n|n-1} \end{array}$$

(8) $$\begin{array}{cc} \; & K_{n}=P_{n-1|n-1}{\left(P_{n-1|n-1}+R\right)}^{-1} \end{array}$$

where I is a unit matrix of 3×3.

### 4.2. Robust Kalman Filter [20]

To remove the outlier, the RKF is applied. Note that although the notation of symbols is the same as with the KF, these elements are different from the KF.

#### 4.2.1. Outlier Correction Using the RKF for Acceleration

Let the state $x={\left[v_{x},v_{y},v_{z},\alpha_{x},\alpha_{y},\alpha_{z}\right]}^{T}$ and the observation $y={\left[\alpha_{x},\alpha_{y},\alpha_{z}\right]}^{T}$. Its state–space model is represented by the following Equation (9).

(9) $$\;\left\{\begin{array}{c} x_{n}=Ax_{n-1}+v_{n}\\ y_{n}=Hx_{n}+w_{n}+z_{n} \end{array}\right.$$

where A is the state-transition matrix shown in Equation (11), $v_{n}$ is the system noise vector and is the six-dimensional Gaussian white noise sequence N(0,Q). Q is a variance–covariance matrix of observation noise and is expressed as follows:

(10) $$\begin{array}{cc} \; & Q=\left[\begin{array}{cccccc} \frac{\Delta t^{3}}{4}\sigma_{x}^{2} & 0 & 0 & \frac{\Delta t^{2}}{2}\sigma_{x}^{2} & 0 & 0\\ 0 & \frac{\Delta t^{3}}{4}\sigma_{y}^{2} & 0 & 0 & \frac{\Delta t^{2}}{2}\sigma_{y}^{2} & 0\\ 0 & 0 & \frac{\Delta t^{3}}{4}\sigma_{z}^{2} & 0 & 0 & \frac{\Delta t^{2}}{2}\sigma_{z}^{2}\\ \frac{\Delta t^{2}}{2}\sigma_{x}^{2} & 0 & 0 & \Delta t\sigma_{x}^{2} & 0 & 0\\ 0 & \frac{\Delta t^{2}}{2}\sigma_{y}^{2} & 0 & 0 & \Delta t\sigma_{y}^{2} & 0\\ 0 & 0 & \frac{\Delta t^{2}}{2}\sigma_{z}^{2} & 0 & 0 & \Delta t\sigma_{z}^{2} \end{array}\right] \end{array}$$

H is the observation matrix shown Equation (12). $w_{n}$ is the observation noise vector and is the three-dimensional Gaussian white noise sequence N(0,R). $z_{n}$ is the outlier vector, R is a variance–covariance matrix of observation noise, the same as Equation (3), and is expressed as follows:

(11) $$\begin{array}{cc} \; & A=\left[\begin{array}{cccccc} 1 & 0 & 0 & \Delta t & 0 & 0\\ 0 & 1 & 0 & 0 & \Delta t & 0\\ 0 & 0 & 1 & 0 & 0 & \Delta t\\ 0 & 0 & 0 & 1 & 0 & 0\\ 0 & 0 & 0 & 0 & 1 & 0\\ 0 & 0 & 0 & 0 & 0 & 1 \end{array}\right] \end{array}$$

(12) $$\begin{array}{cc} \; & H=\left[\begin{array}{cccccc} 0 & 0 & 0 & 1 & 0 & 0\\ 0 & 0 & 0 & 0 & 1 & 0\\ 0 & 0 & 0 & 0 & 0 & 1 \end{array}\right] \end{array}$$

The state estimation of Equation (9) can be completed by the algorithm of the RKF shown in Equations (13)–(20).

[One-step-ahead prediction]

(13) $$\begin{array}{cc} \; & {\hat{x}}_{n|n-1}=A{\hat{x}}_{n-1|n-1} \end{array}$$

(14) $$\begin{array}{cc} \; & P_{n|n-1}=AP_{n-1|n-1}A^{T}+Q \end{array}$$

[Filtering]

(15) $$\begin{array}{cc} \; & {\hat{x}}_{n|n}={\hat{x}}_{n|n-1}+L\left(e_{n}-{\hat{z}}_{n}\right) \end{array}$$

(16) $$\begin{array}{cc} \; & P_{n|n}=\left(I-\mathrm{LH}\right)P_{n|n-1} \end{array}$$

(17) $$\begin{array}{cc} \; & L=P_{n|n-1}H^{T}{\left(\sigma_{n}^{2}\right)}^{-1} \end{array}$$

(18) $$\begin{array}{cc} \; & e_{n}=y_{n}-H{\hat{x}}_{n|n-1} \end{array}$$

(19) $$\begin{array}{cc} \; & {\hat{z}}_{k,n}=\left\{\begin{array}{cc} e_{k_{n}}-\sigma_{kk_{n}} & if\;e_{k_{n}}\geq \sigma_{kk_{n}}\\ e_{k_{n}}+\sigma_{kk_{n}} & if\;e_{k_{n}}<-\sigma_{kk_{n}}\\ 0 & otherwise \end{array}\right.\left(k=x,y,z\right) \end{array}$$

(20) $$\begin{array}{cc} \; & \sigma_{n}^{2}=HP_{n|n-1}H^{T}+R \end{array}$$

where I is a unit matrix of 6×6. Since the ${\hat{z}}_{k_{n}}$ is independent of other random variables, $\sigma_{kk_{n}}$ can be regarded as depending on the square root of the diagonal term of $\sigma_{kk}^{2}$ in Equation (3) [21]. Furthermore, the evaluation of integration errors is performed by using the time derivative of pressure, dP/dt.

(21) $$\begin{array}{cc} \; & \frac{dP}{dt}≃\frac{P_{n}-P_{n-1}}{\beta \Delta t} \end{array}$$

(22) $$\begin{array}{cc} \; & v_{n}^{s}=\left\{\begin{array}{cc} 2v_{n}^{s}\frac{|dP/dt|}{|v_{z_{n}}^{s}|} & if\;|v_{z_{n}}^{s}|-\frac{dP}{dt}\geq 0.1\\ v_{n}^{s} & otherwise \end{array}\right. \end{array}$$

where β is a calibration coefficient 1025.0×9.8.

#### 4.2.2. Magnetic Disturbance Correction Using the RKF for Geomagnetic Sensors

Let the state $x={\left[m_{x},m_{y},m_{z}\right]}^{T}$ and the observation $y={\left[m_{x},m_{y},m_{z}\right]}^{T}$. Its state–space model is represented by the following Equation (23).

(23) $$\;\left\{\begin{array}{c} x_{n}=x_{n-1}+v_{n}\\ y_{n}=x_{n}+w_{n}+z_{n} \end{array}\right.$$

where $v_{n}$ is the system noise vector and is the three-dimensional Gaussian white noise sequence N(0,Q). $Q_{n}$ is the variance–covariance matrix of the system noise is determined according to the amount of rotation of ψ, which means that the corrections for rotating and stationary states are different, so it is expressed as Equation (24) as follows:

(24) $$\begin{array}{cc} \; & Q_{n}=\left\{\begin{array}{cc} \frac{\Delta t^{2}}{2}\sigma_{n-1}^{2} & if\;|\omega_{z_{n-1}}-\omega_{z_{n}}|\;\leq \;0.02\\ \frac{3}{2}\sigma_{n-1}^{2} & otherwise \end{array}\right. \end{array}$$

where $\sigma_{n-1}^{2}$ is calculated using Equation (32). $w_{n}$ is the observation noise vector and is the three-dimensional Gaussian white noise sequence N(0,R). $z_{n}$ is the outlier vector, R is a variance–covariance matrix of observation noise, which is the same as Equation (3). The geomagnetic field exhibits significant variations depending on the location; therefore, in this paper, it is treated as a time-varying system. Specifically, the conditional variance–covariance of the state x is assumed to change with time. That is, in the one-step-ahead prediction process, the variance–covariance matrix of the system noise is adjusted according to the state with time, as shown in Equation (24). The state estimation of Equation (23) can be completed by the algorithm of the RKF shown in Equations (25)–(32).

[One-step-ahead prediction]

(25) $$\begin{array}{cc} \; & {\hat{x}}_{n|n-1}=A{\hat{x}}_{n-1|n-1} \end{array}$$

(26) $$\begin{array}{cc} \; & P_{n|n-1}=P_{n-1|n-1}+Q_{n} \end{array}$$

[Filtering]

(27) $$\begin{array}{cc} \; & {\hat{x}}_{n|n}={\hat{x}}_{n|n-1}+L\left(e_{n}-{\hat{z}}_{n}\right) \end{array}$$

(28) $$\begin{array}{cc} \; & P_{n|n}=\left(I-L\right)P_{n|n-1} \end{array}$$

(29) $$\begin{array}{cc} \; & L=P_{n|n-1}{\left(\sigma_{n}^{2}\right)}^{-1} \end{array}$$

(30) $$\begin{array}{cc} \; & e_{n}=y_{n}-{\hat{x}}_{n|n-1} \end{array}$$

(31) $$\begin{array}{cc} \; & {\hat{z}}_{k,n}=\left\{\begin{array}{cc} e_{k_{n}}-\sigma_{kk_{n}} & if\;e_{k_{n}}\geq \sigma_{kk_{n}}\\ e_{k_{n}}+\sigma_{kk_{n}} & if\;e_{k_{n}}<-\sigma_{kk_{n}}\\ 0 & otherwise \end{array}\right.\left(k=x,y,z\right) \end{array}$$

(32) $$\begin{array}{cc} \; & \sigma_{n}^{2}=P_{n|n-1}+R \end{array}$$

**Figure 4:**
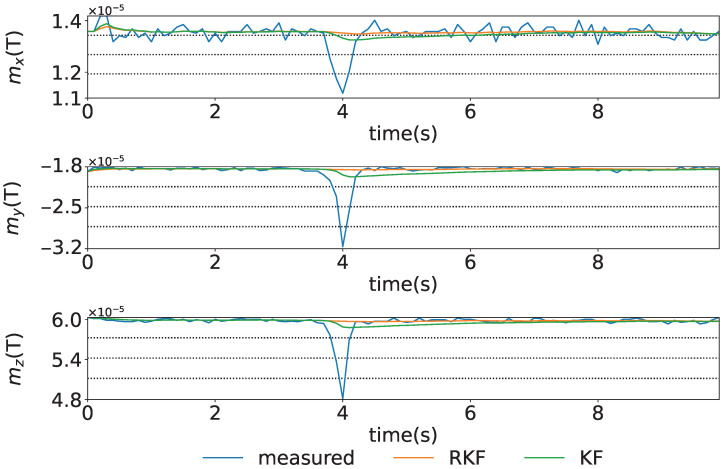
Time series of magnetic disturbances intentionally generated while stationary: x,y, and *z* axes shown from top to bottom.

A time series of intentionally generated magnetic disturbances in stationary conditions to verify the magnetic disturbance correction of the geomagnetic sensors using KF and RKF is shown in Figure 4. The figure shows the blue, orange, and green lines representing the measured data, the estimated states by the RKF, and the estimated states by the KF, respectively. From this figure, it can be seen that the magnetic disturbance that occurred around 4 s has been removed using the RKF. Compared to the KF, the RKF is more effective in eliminating spike-like magnetic disturbances and achieves stabilization more quickly.

**Figure 5:**
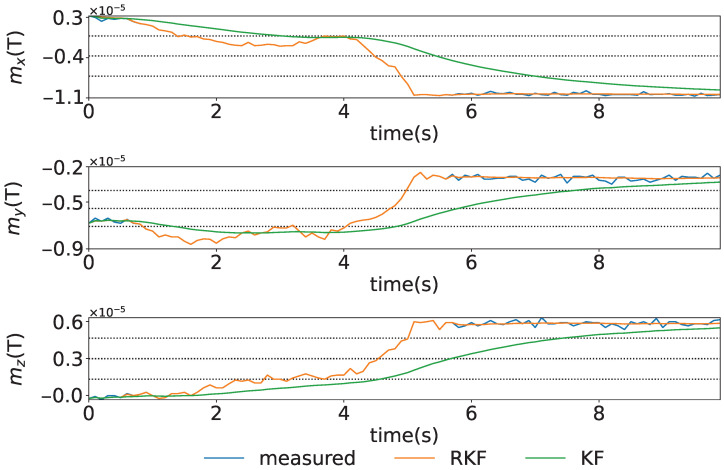
The time series for the case where the geomagnetic sensor is rotated on the x–y plane in the S-frame and then held stationary under conditions of no magnetic disturbance. The figure shows the x,y, and *z* axes from the top to bottom.

The time series for the case where the geomagnetic sensor is rotated on the x–y plane in the S-frame and then held stationary under conditions of no magnetic disturbance is shown in Figure 5. The figure shows the blue, orange, and green lines representing the measured, the estimated states by the RKF, and the estimated states by the KF, respectively. In this study, the variance–covariance Q of the system noise is determined according to the amount of rotation of ψ, which means that the corrections for rotating and stationary states are different. As a result, more effective state estimation is achieved compared to the KF. In addition, since the system is treated as a time-varying system, the state estimation performs better under transient conditions than when using the conventional KF.

### 4.3. Quaternion-Based Coordinate Transformation

Converting to quaternions can be expressed as follows [22].

(33) $$\left[\begin{array}{c} q_{0_{n}}\\ q_{1_{n}}\\ q_{2_{n}}\\ q_{3_{n}} \end{array}\right]=\left[\begin{array}{c} \cos\frac{\phi_{n}^{s}}{2}\cos\frac{\theta_{n}^{s}}{2}\cos\frac{\psi_{n}^{s}}{2}+\sin\frac{\phi_{n}^{s}}{2}\sin\frac{\theta_{n}^{s}}{2}\sin\frac{\psi_{n}^{s}}{2}\\ \sin\frac{\phi_{n}^{s}}{2}\cos\frac{\theta_{n}^{s}}{2}\cos\frac{\psi_{n}^{s}}{2}-\cos\frac{\phi_{n}^{s}}{2}\sin\frac{\theta_{n}^{s}}{2}\sin\frac{\psi_{n}^{s}}{2}\\ \cos\frac{\phi_{n}^{s}}{2}\sin\frac{\theta_{n}^{s}}{2}\cos\frac{\psi_{n}^{s}}{2}+\sin\frac{\phi_{n}^{s}}{2}\cos\frac{\theta_{n}^{s}}{2}\sin\frac{\psi_{n}^{s}}{2}\\ \cos\frac{\phi_{n}^{s}}{2}\cos\frac{\theta_{n}^{s}}{2}\sin\frac{\psi_{n}^{s}}{2}-\sin\frac{\phi_{n}^{s}}{2}\sin\frac{\theta_{n}^{s}}{2}\cos\frac{\psi_{n}^{s}}{2} \end{array}\right]$$

The conversion from quaternions to Euler angles is performed by the following equation [23].

(34) $$\left[\begin{array}{c} \phi \\ \theta \\ \psi \end{array}\right]=\left[\begin{array}{cc} \tan^{-1}\frac{q_{3}^{2}-q_{2}^{2}-q_{1}^{2}+q_{0}^{2}}{2(q_{0}q_{1}+q_{2}q_{3})} & \left(-\pi /2<\phi <\pi /2\right)\\ \sin^{-1}\left\{2\left(q_{1}q_{3}-q_{0}q_{2}\right)\right\} & \left(-\pi /2<\theta <\pi /2\right)\\ \tan^{-1}\frac{q_{1}^{2}+q_{0}^{2}-q_{3}^{2}-q_{2}^{2}}{2(q_{1}q_{2}+q_{0}q_{3})} & \left(-\pi \leq \psi \leq \pi \right) \end{array}\right]$$

Note that the range of ϕ and θ depends on $\phi^{\alpha }$ and $\theta^{\alpha }$.

### 4.4. Coordinate Transformation to the T-Frame

The coordinate transformation to the T-frame is expressed by rotating *ψ*_*n*_ by 90° as follows.

(35) $$\;\left[\begin{array}{c} p_{x_{n}}^{t}\\ p_{y_{n}}^{t} \end{array}\right]=\left[\begin{array}{c} p_{x_{n-1}}^{t}+\Delta tV_{n}\sin\left(\psi_{n}\right)\epsilon \\ p_{x_{n-1}}^{t}+\Delta tV_{n}\cos\left(\psi_{n}\right)\epsilon \end{array}\right]$$

where ϵ is a calibration coefficient 1.5 in this paper. Here, a resultant velocity $V_{n}$ can be calculated by the following equation.

(36) $$\begin{array}{cc} \; & V_{n}=\sqrt{{v_{x_{n}}^{s}}^{2}\beta +{v_{y_{n}}^{s}}^{2}\left(1-\beta \right)}\gamma \end{array}$$

(37) $$\begin{array}{cc} \; & \gamma =\left\{\begin{array}{cc} \frac{0.35}{V_{n}} & if\;V_{n}\geq 0.35\\ 1 & otherwise \end{array}\right. \end{array}$$

where β is a weight coefficient 0.7 in this paper.

### 4.5. Waypoint Configuration

To reduce the accumulation of integration errors, the azimuth and distance to the nearest waypoint are calculated using the following equation. In addition, it always continues to search for the nearest point by conditional branching. here, $p_{n}^{e}$ and $\psi_{n}^{G}$ are displayed for the diver to navigate.

(38) $$\begin{array}{cccc} \; & \left[\begin{array}{c} p_{x_{n}}^{g}\\ p_{y_{n}}^{g} \end{array}\right] & = & \left[\begin{array}{c} p_{x_{i}}^{w}-p_{x_{n}}^{t}\\ p_{y_{i}}^{w}-p_{y_{n}}^{t} \end{array}\right] \end{array}$$

(39) $$\begin{array}{cc} \; & \psi_{n}^{l}=-\tan^{-1}\frac{p_{y_{n}}^{g}}{p_{x_{n}}^{g}}\;\left(-\pi \leq \psi_{n}^{l}\leq \pi \right) \end{array}$$

(40) $$\begin{array}{cc} \; & \psi_{n}^{G}=\psi_{n}^{l}-\psi_{n}+\frac{\pi }{2} \end{array}$$

(41) $$\begin{array}{cc} \; & p_{n}^{e}=\sqrt{{p_{x_{n}}^{g}}^{2}+{p_{y_{n}}^{g}}^{2}} \end{array}$$

## 5. Experimental Results and Discussions

### 5.1. Experimental Results and Discussions of Attitude Estimation

**Figure 6:**
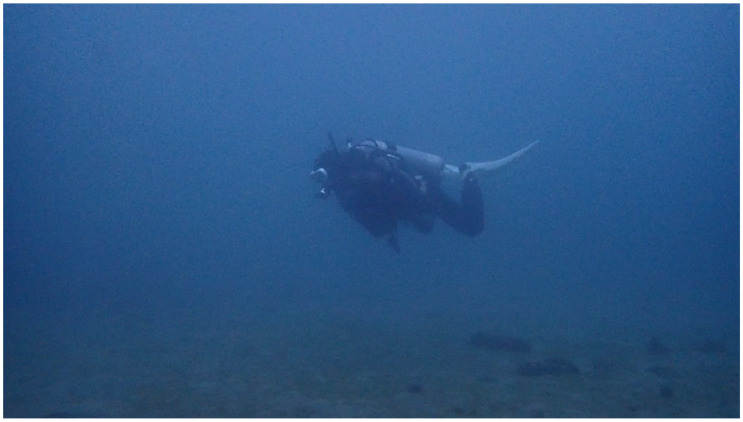
Moving diver.

The diver’s movements were recorded, and the periodicity of the motion was verified. An example is shown in Figure 6. Within 14 s, five fin kicks were observed, indicating that the diver’s posture changes at a frequency of approximately 0.36 Hz.

**Figure 7:**
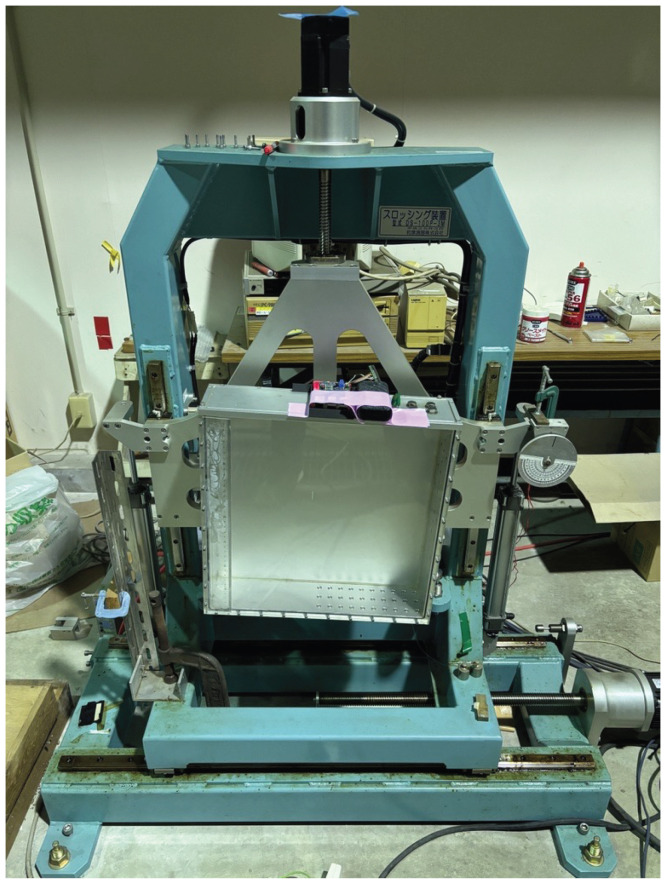
Overview of forced oscillator.

To verify the accuracy of attitude estimation with the proposed system, a forced oscillator (DS-100P-3M) manufactured by Izumi Instruments Co., Ltd. (Tokyo, Japan), as shown in Figure 7, was used. The device was set to vibrate at *ϕ* ± 5° and a frequency of 0.35 Hz for the verification.

**Figure 8:**
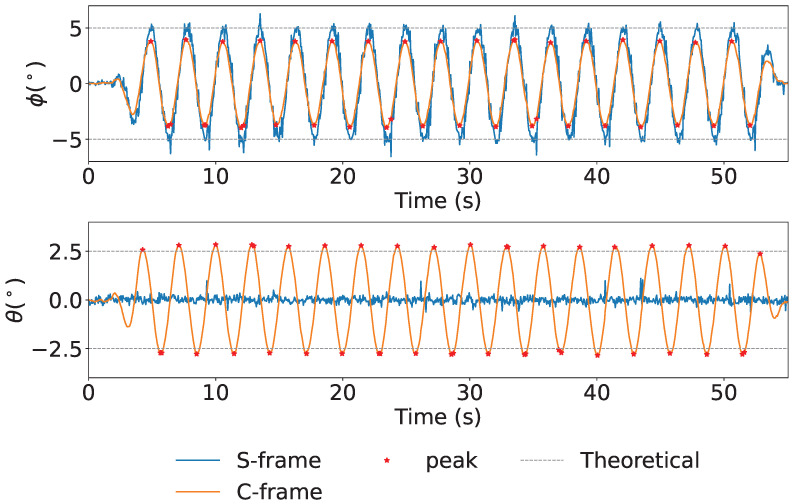
Attitude: The figure shows the ϕ and θ axes from the top down.

**Figure 9:**
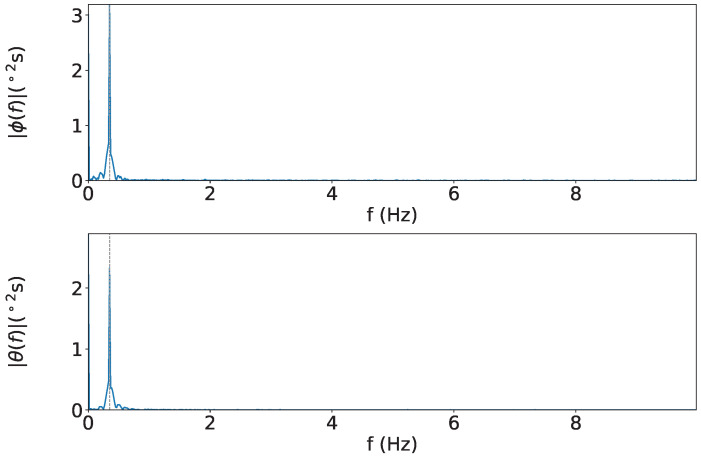
Spectra: The figure shows the ϕ axis on top and θ axis on the bottom.

The time series data of attitude angles, and the spectrum of the attitude angles are shown in Figure 8 and Figure 9, respectively. The time series of attitude angles in Figure 8, blue line, orange line, red dots, and gray line represent the S-frame, C-frame, peak value, and theoretical value, respectively. It shows that the *ϕ* angle, corrected using the KF applied to the data obtained from the accelerometer, removes the spike noise from acceleration. The average RMS was ±1.23° for *ϕ* and ±0.26° for *θ*. The spectrum of the attitude is shown in Figure 9, the gray dotted line in the figure indicates 3 Hz. From Figure 9 it can be confirmed that the motion at the set frequency was successfully measured.

### 5.2. Experimental Results and Discussions of the Underwater Environment

**Figure 10:**
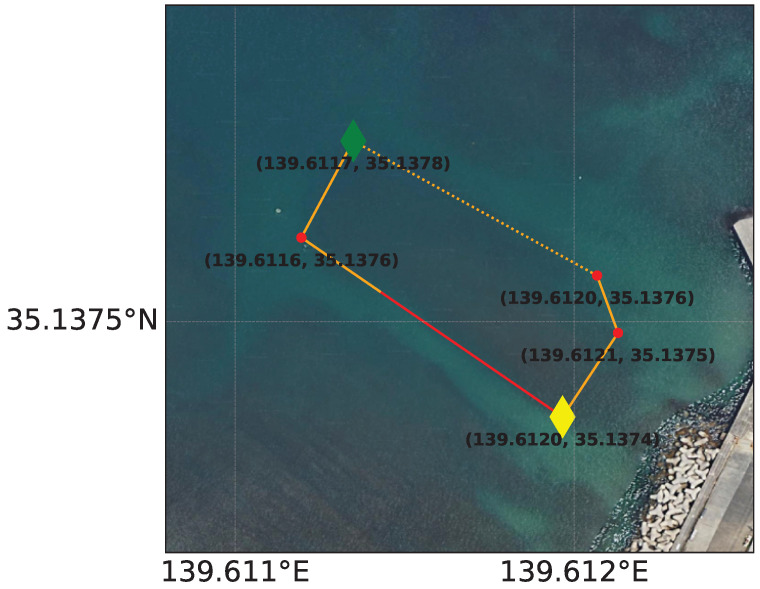
Kajinohama beach [24].

**Figure 11:**
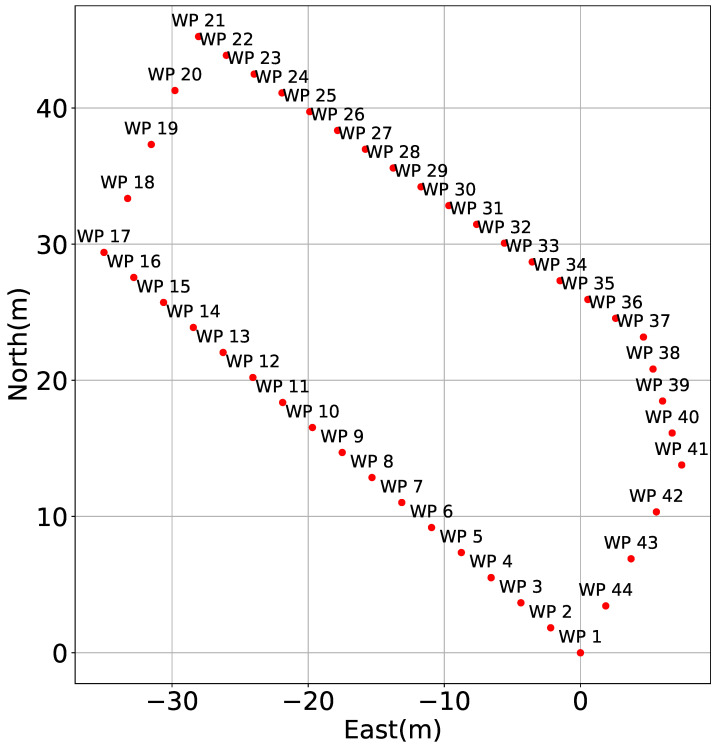
Waypoint numbered from WP1-44 [25].

**Figure 12:**
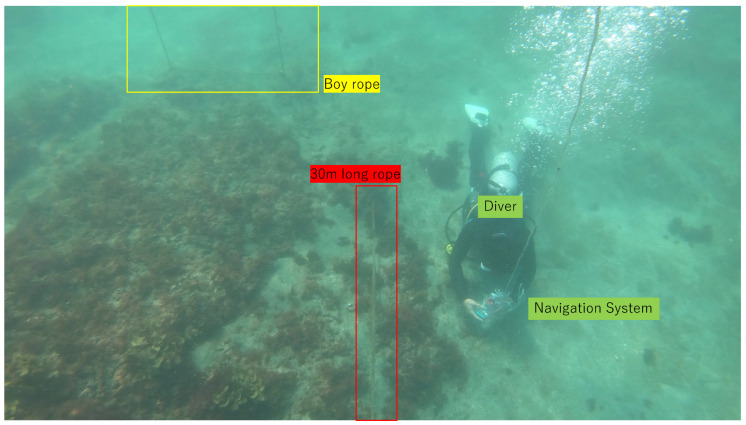
Measurement start and end points.

**Figure 13:**
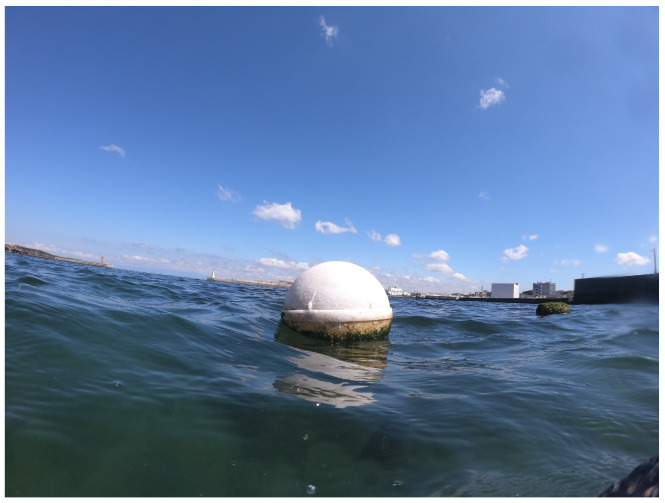
Buoy tied to the bottom of the sea [25].

**Figure 14:**
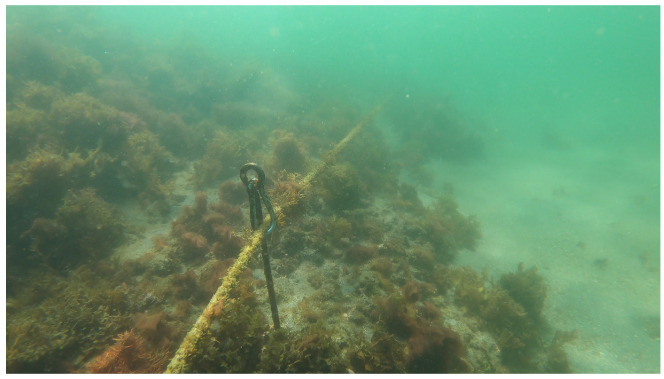
A 30 m long rope stretched along a rock in the sea (red line in Figure 10) [25].

The experiment was conducted at Kajinohama beach in Joga Shima, Miura City, Kanagawa, Japan, on 26 July 2024. The visibility was about 5 m, and the sea conditions were swell caused by the typhoon. Waypoints are set on the red and orange lines in Figure 10. The waypoint set that latitude and longitude in the WGS84 coordinate system transformed into the T-frame. Points were added by iterative calculations so that the distance was within 5 m and numbered from WP1-44, as shown in Figure 11. The yellow point in Figure 10 is the measurement start and end point shown in Figure 12, which can be seen at the buoy tied from the bottom of the sea, as shown in Figure 13, and from the end of a rope shown in the red line in Figure 10 and Figure 14. The proposed system was held in front of the diver’s line of sight, and the route was determined by the system’s instructions. For verification, a buoy was equipped with a GNSS receiver and tied to the diver, and position information on the sea surface was recorded simultaneously. The evaluation of the results was based on the following neighborhoods that could be passed.

**Figure 15:**
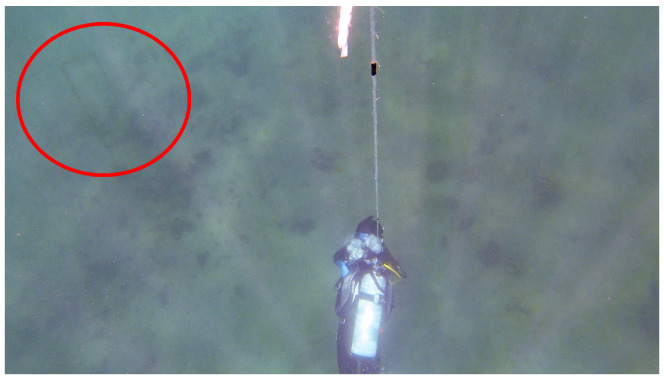
Underwater structure (green point in Figure 10), as indicated by the red circle in the figure [25].

- A 30 m long rope stretched along a rock in the sea (red line in Figure 10 and Figure 14);
- Underwater structure (green point in Figure 10 and Figure 15);
- Underwater reefs (orange dots in Figure 10).

**Figure 16:**
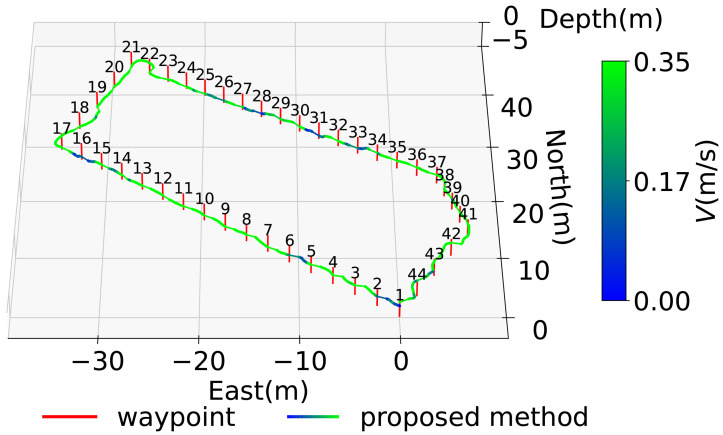
Waypoint and diving trajectory.

**Figure 17:**
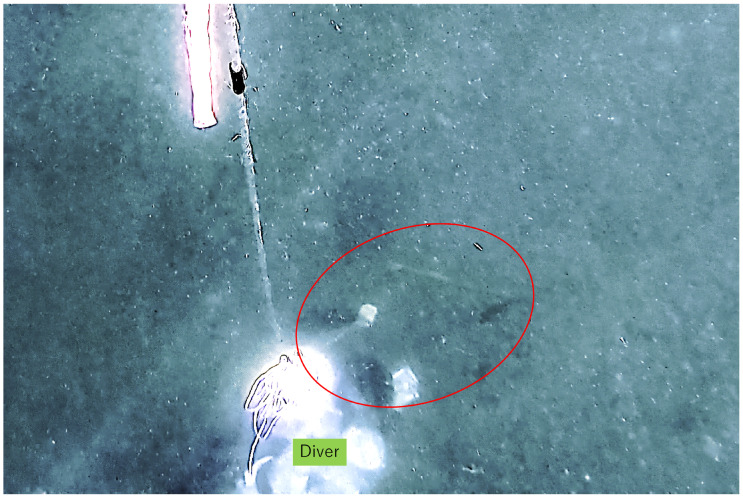
Underwater structure near the green point in Figure 10, as indicated by the red circle in the figure.

**Figure 18:**
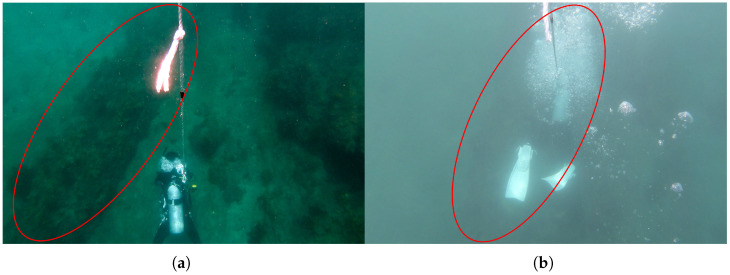
Underwater reefs WP21–32, as indicated by the red circle in the figure: (**a**) under high water clarity conditions. (**b**) during the experiment.

In Figure 16, the diver’s trajectory illustrates the path taken while sequentially following the waypoints indicated by red lines. The *z*-axis in the figure represents depth, allowing for visualization of vertical movement during the dive. The trajectory generated by the proposed system is shown using a color gradient from blue to green, where the color represents the magnitude of the resultant velocity *V* (Equation (36)). Even when decelerating due to direction changes, the estimated speed does not diverge due to the correction based on water pressure. Where speeds are reduced, it is considered that this is due to turning or sea conditions. Quaternion-based attitude estimation was able to suppress the attitude angle drift error of the gyro sensor, as the error was evaluated in the S-frame and corrected using the KF. However, since ϕ and θ are constrained between −π/2 and π/2, future work should aim to extend the measurable range to −π to π. Additionally, the RKF applied to geomagnetic sensors was unaffected by gyro drift and enabled more detailed evaluation of geomagnetic sensor errors compared to the previous study [16]. The reason for the inflation from WP21-22 in Figure 16 is that deceleration was recorded during turning from WP17-18 but not recorded during turning from WP21-22. As a prospect, it is necessary to improve the threshold value set in Equation (22) or to calculate the velocity by coordinate transformation from the position in the S-frame without relying on the resultant velocity. However, the aforementioned evaluation criteria are generally met as shown in Figure 17 and Figure 18. Therefore, the proposed system is considered to have the desired accuracy. As a result, the proposed system has the advantage of being robust against magnetic disturbances caused by diving equipment and is resilient to wave conditions comparable to those observed during the experiments. On the other hand, since the system relies on resultant velocity, it has a limitation in that it cannot provide accurate navigation when the diver is drifting.

**Figure 19:**
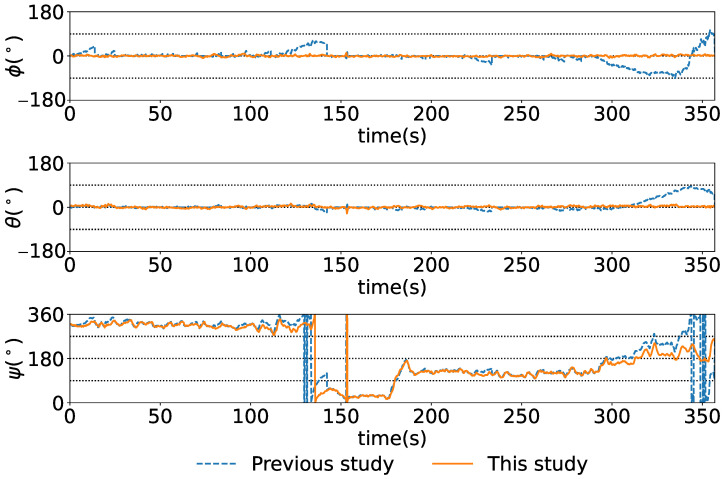
Attitude of ϕ, θ, and ψ from the top to the bottom.

**Figure 20:**
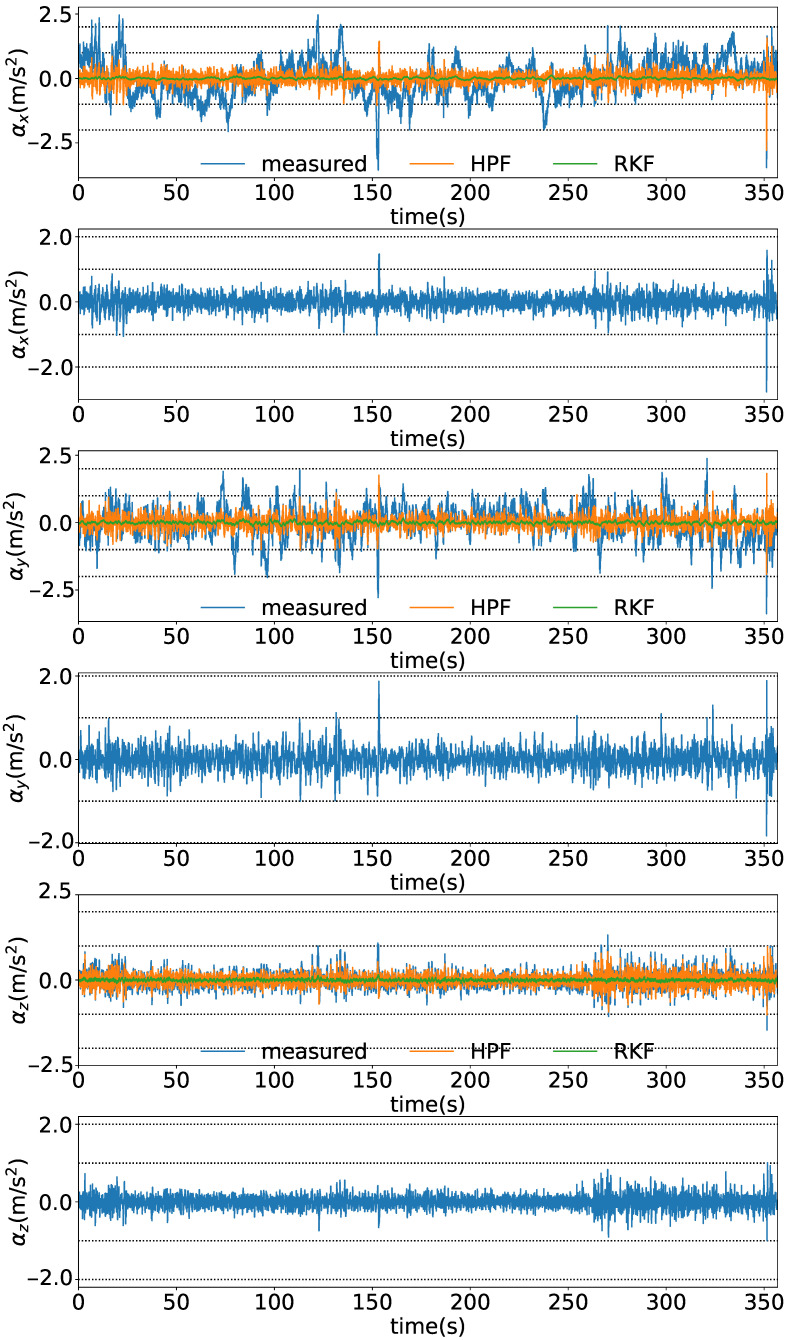
Time series of the acceleration sensor. The figure shows from the top to the bottom, the time series of the *x* axis, the time series of the noise component of the *x* axis, the time series of the *y* axis, the time series of the noise component of the *y* axis, the time series of the *z* axis, and the time series of the noise component of the *z* axis from.

**Figure 21:**
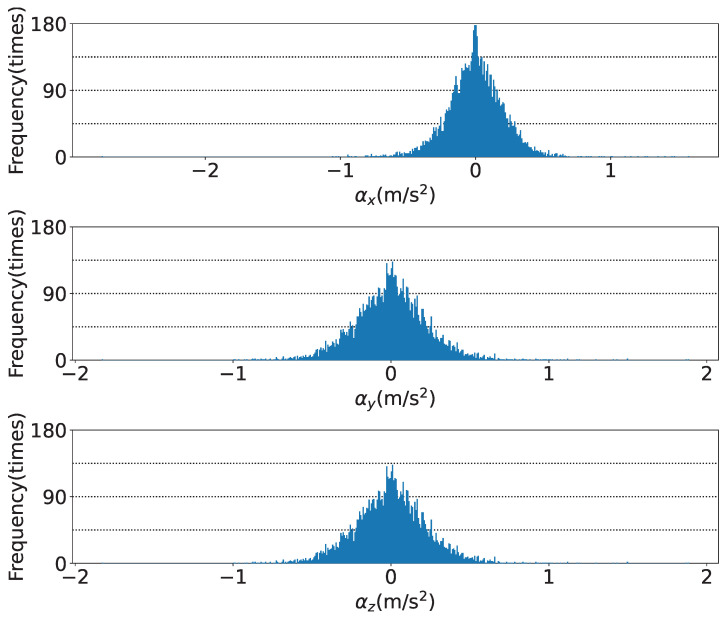
Histogram of the acceleration noise component: *x*, *y*, and *z* axes from the top.

Figure 19, Figure 20 and Figure 21 show the time series of the attitude angles, the acceleration sensor, and the histogram of the acceleration noise component, respectively. In the time series of the attitude in Figure 19, the blue and orange lines represent the previous study method and this study method, respectively. To simulate the previous study, the analog attitude estimator was activated, with $\psi_{n}^{a}$ and $\theta_{n}^{a}$ indicated in ±1°. These figures show that drift errors are more effectively removed compared to the previous method. The time series of the acceleration sensor in Figure 20 shows the *x*, *y*, and *z* axis data, along with the noise components between the HPF and the RKF for each axis. The blue, orange, and green lines represent the measured values offset by the average, the values filtered by the HPF, and the estimated states by the RKF, respectively. These figures show that outliers are successfully removed. The noise component time series between the HPF and the RKF for the *x*, *y*, and *z* axes demonstrates that the RKF effectively removes pulse-like outliers that occur when the diver kicks out. The histogram of the acceleration noise component in Figure 21 shows that, consistent with the filter design, the noise residual follows a Gaussian distribution.

**Figure 22:**
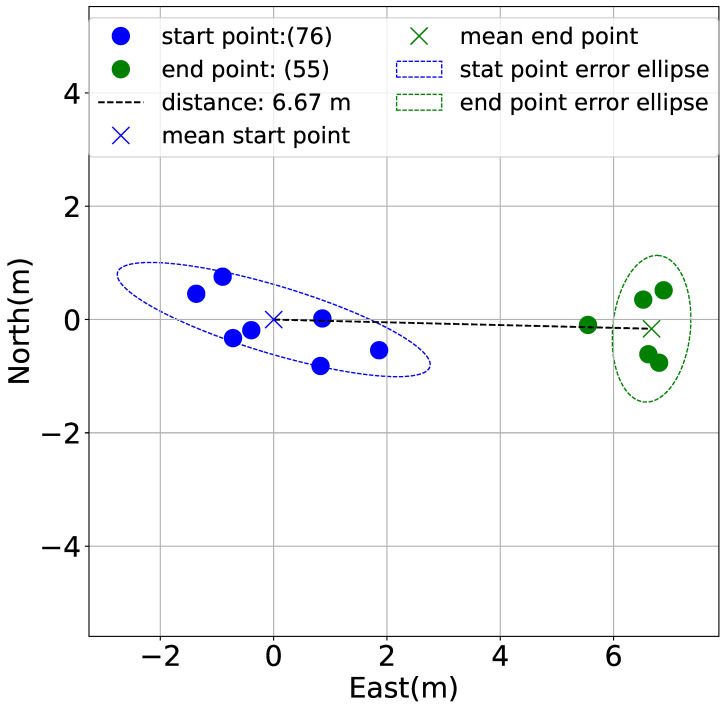
GNSS signals at the start and end point [25].

Guidance was recorded from WP44 to the start point in Figure 16, but in reality, it was a little further away. The difference is due to an offset error caused by the ocean current and the calibration of the geomagnetic sensor. In order to make a quantitative assessment, a buoy equipped with a GNSS receiver was tension moored at the start and end points. The GNSS receiver is built into a smartphone (iPhone 13 manufactured by Apple Inc. (Cupertino, California, United States) ), records using the Precise Point Positioning (PPP) method, and the obtained signal is utilized without any signal processing. In Figure 22, the average of the GNSS signal values at each point was calculated to obtain the distance between the start and end points. The GNSS signals measured at the start point (76 samples) are indicated by blue circles, the average values are indicated by blue × signs, and error ellipses are indicated by the blue dotted line. The GNSS signals measured at the end point (55 samples) are indicated by green circles, the mean value is indicated by green × signs and error ellipses are indicated by the green dotted line. Note that to evaluate the error of the GNSS receiver around the stationary position, measurements were taken while stationary on the ground for one hour. The error ellipse had a width of 1.627 m and a height of 1.780 m. The GNSS signal, therefore, has the desired accuracy. A straight line connecting two × marks was 6.67 m. Thus, the start point can be seen when visibility is more than 7 m. Even when visibility is less than 7 m, the start point can be seen if the diver surfaces at the end point, which suggests that the diver can return to the start point.

## 6. Conclusions

In this study, we developed an underwater navigation system using low-cost sensors that enables divers to be guided along a predefined route from the start point to the goal point. We extended the previous study [16], which replaced the analog attitude estimator with the sensor fusion attitude estimation method. As a result, forced oscillator trials achieved pitch and roll RMS errors of ±1.23° and ±0.26°, respectively. In field experiments, divers were able to return to the start point with an average error of 6.67 m. This indicates that the start point can be visually identified when visibility exceeds 7 m. Even when visibility is below 7 m, the start point can be located if the diver surfaces at the end point, suggesting that return navigation is feasible. Therefore, the system demonstrates practical applicability. Moreover, the field experiments showed that the proposed system is robust against magnetic disturbances caused by diving equipment and resilient to wave conditions comparable to those observed during the experiments. This result not only helps prevent incidents where semi-professional divers with 100–500 dives fail to return, but also avoids safety-compromising factors found in conventional navigation equipment. Therefore, it demonstrates the potential to contribute significantly to improving the safety of diving activities. As a result, it became possible to show the following.

1. The estimation of velocity can be achieved even when employing low-cost sensors. Due to the substantial pressure variations present underwater, depth changes are measured using a water pressure gauge to correct integration errors from acceleration to velocity.
2. The proposed system addresses both cost and usability in restricted environments. Furthermore, as it does not depend on external information such as GNSS or sonar, it eliminates the necessity for large-scale equipment and tethering cables to the water surface, thereby significantly enhancing the safety of underwater operations.
3. The estimation of attitude can be achieved even in circumstances involving the utilization of low-SNR sensors. Because preprocessing of the accelerometer and geomagnetic sensor signals is collected using the RKF, sensor fusion is performed in the S-frame using the KF. Finally, the estimated attitude is transformed into the C-frame using quaternions.
4. The proposed method demonstrates a capacity for precise azimuth estimation in environments characterized by magnetic disturbances, thereby exhibiting superior performance in comparison to the conventional KF when confronted with transient conditions. This is due to the implementation of the RKF, which models the geomagnetic sensor as a time-varying system and adapts system noise according to changes in the gyro sensor’s *z* axis.
5. The present paper utilizes a quaternion-based attitude estimation that functions independently of the Euler angle rotation matrix. This approach helps to avoid gimbal lock because signals are processed in the S-frame and converted to quaternions. Subsequently, the quaternion exclusively undergoes a transformation into Euler angles to display the azimuth angle to the user.

Finally, the following challenges remain to be addressed in future work:

1. The quaternion-based attitude estimation is currently constrained such that ϕ and θ are limited to the range from −π/2 to π/2. Since divers sometimes look upward toward the water surface, it is necessary to expand the measurable range to −π to π.
2. Regarding localization, when using composite velocity, accurate positioning cannot be achieved in drift states where the main flow velocity component and the diver’s facing direction do not align. It is necessary to perform localization on the T-frame by using the estimated velocity in the S-frame and the azimuth angle in the C-frame.

## Data Availability

Dataset available on request from the authors. The raw data supporting the conclusions of this article will be made available by the authors on request.

## Abbreviations and Nomenclature

The following abbreviations are used in this manuscript:

T-frame	True frame
C-frame	Computer frame
S-frame	Sensor frame
KF	Kalman filter
RKF	Robust Kalman filter
SSF	Sensitivity Scale Factor
HPF	High-pass filter
LPF	Low-pass filter
RMS	Root Mean Square
$\alpha_{n}$	Acceleration in S-frame ($m/s^{2}$)
$\omega_{n}$	Angular velocity in S-frame (rad/s)
$m_{n}^{s}$	Magnetic field in S-frame (G)
$v_{n}^{s}$	Velocity in S-frame (m/s)
$V_{n}$	Resultant velocity (m/s)
$p_{n}^{t}$	Position in T-frame (m)
$p_{i}^{w}$	Preset waypoint (m)
$p_{n}^{g}$	Calculated distance to waypoint (m)
$P_{n}$	Pressure (Pa)
$q_{n}$	Quaternion
$\phi_{n}^{s}$	Roll motion in S-frame from KF (rad)
$\phi_{n}^{\alpha }$	Roll motion from accelerometer (rad)
$\phi_{n}$	Roll motion in C-frame (rad)
$\theta_{n}^{s}$	Pitch motion in S-frame from KF (rad)
$\theta_{n}^{\alpha }$	Pitch motion in from accelerometer (rad)
$\theta_{n}$	Pitch motion in C-frame (rad)
$\psi_{n}^{s}$	Heading angle in S-frame from KF (rad)
$\psi_{n}^{m}$	Heading angle from geomagnetic sensor (rad)
$\psi_{n}$	Heading angle in C-frame (rad)
Δ t	Sampling interval (s)
